# Rare Jejunal Large B-cell Lymphoma Mimicking a Crohn’s Disease Without Terminal Ileum Involvement

**DOI:** 10.7759/cureus.26450

**Published:** 2022-06-30

**Authors:** Alexander J Kaye, Catherine Choi, Vincent Wong, Weizheng Wang

**Affiliations:** 1 Internal Medicine, Rutgers University New Jersey Medical School, Newark, USA; 2 Gastroenterology and Hepatology, Rutgers University New Jersey Medical School, Newark, USA; 3 Internal Medicine-Pediatrics, Rutgers University New Jersey Medical School, Newark, USA

**Keywords:** dlbcl, diffuse large b-cell lymphoma, inflammatory bowel disease, crohn`s disease, jejunum, non-hodgkin lymphoma (nhl)

## Abstract

This case describes a 49-year-old man who presented with a several-month history of melena, and unintentional weight loss. Prior esophagogastroduodenoscopy and colonoscopy were unrevealing. Further evaluation with capsule endoscopy showed patchy erythematous mucosa in the jejunum creating suspicion for Crohn’s Disease. Subsequent push enteroscopy found nodular and congested patchy mucosa of jejunum, and stigmata of bleeding in the proximal and mid-jejunum. Repeat colonoscopy showed a diffuse area of erythematous mucosa in the recto-sigmoid colon, and moderately congested mucosa in the ascending colon, but a normal terminal ileum. A small bowel biopsy eventually revealed large B-cell lymphoma. This is one of the first seven reported cases of small bowel lymphoma mimicking Crohn’s Disease and the first to not have any ileal involvement.

## Introduction

Obscure gastrointestinal bleed (GB), defined as a GB without an identifiable source despite esophagogastroduodenoscopy and colonoscopy, represents five percent of all GB events [1]. The mortality associated with obscure GB has been noted to be 12%, therefore identifying the source of bleeding can be lifesaving [2]. The likelihood of a given obscure GB etiology varies with age. Patients under the age of 40 years old are more prone to intestinal tumors, polyps, a Meckel’s diverticulum, a Dieulafoy lesion, and inflammatory bowel disease (IBD) [1]. On the other hand, patients who are older than 40 years old with obscure GB most often have vascular lesions, which represent 40% of obscure GBs in this age group, or nonsteroidal anti-inflammatory drug-induced small bowel disease [1]. Approximately 75% of obscure GBs originate from the small bowel [1].

Primary small bowel tumors, a concerning potential cause of obscure GB, account for one to three percent of all gastrointestinal malignancies [3]. In the year 2014, it was estimated that 9,160 patients were diagnosed with a small bowel malignancy [3]. Higher risk for small bowel lymphoma exists in those who are: male, age 50-69 years old, black, use alcohol or tobacco, are obese, or eat a high-fat diet [3]. Symptomatology associated with small bowel malignancy can vary but can include weight loss, abdominal pain, nausea, vomiting, and anemia [3]. The average interval between the onset of symptoms and a diagnosis of small bowel malignancy is 10 months [3]. There sometimes can be a delay in the diagnosis of a gastrointestinal malignancy due to presentations that mimic IBD [4]. This case describes a 49-year-old man who presented with significant weight loss, and recurrent melena in the setting of obscure GB whose workup was notable for new erythema and mucosal congestion in several locations along the gastrointestinal tract.

## Case presentation

A 49-year-old African American man with a past medical history of atrial flutter, hypertension, iron deficiency anemia, chronic nonocclusive right femoral deep vein thrombosis, and recent pulmonary embolisms was referred to the emergency department for a hemoglobin of 6.4 g/dL on routine blood work. Review of systems was positive for four months of intermittent melena, 40 pounds of unintentional weight loss over six months, palpitations, and fatigue. The social history was positive for smoking with a 20-pack-year history. His outpatient medications were pertinent for apixaban, ferrous sulfate, pantoprazole, and metoprolol succinate.

The patient was recently hospitalized for bilateral pulmonary embolisms, which were diagnosed by a CT of the chest. An abdomen/pelvis CT at the same time revealed intramural thickening of jejunal bowel loops with mesenteric chain adenopathy and adjacent reactive perienteric and perisplenic ascites. At the time of this imaging, no additional abdominal workup was pursued. He had also been hospitalized two months prior for symptomatic anemia. During that hospitalization, the patient underwent a colonoscopy revealing diverticulosis, internal and external hemorrhoids, none of which were bleeding, as well as an esophagogastroduodenoscopy which identified a non-bleeding subepithelial esophageal mass found on pathology to be fibroconnective tissue with mild chronic inflammation and rare admixed small nerve bundles. Admission laboratory serum studies were notable for the findings in Table 1.

**Table 1:** Notable Emergency Department Laboratory Serum Studies INR: International normalized ratio; PTT: Partial thromboplastin time.

Name of laboratory study (unit)	Value of laboratory study	Range of normal values
White blood cell count (per µL)	8,000	4,000 - 11,000
Hemoglobin (g/dL)	6.4	14.0 - 18.0
Hematocrit (%)	19.3	42.0 - 54.0
Platelets (per µL)	453,000	150,000 - 450,000
Reticulocyte count (%)	5.8	0.5 - 2.0
INR	1.2	0.90 - 1.2
PTT (seconds)	156.7	24.0 - 34.2
Lactate dehydrogenase (U/L)	456	120 - 250
Total iron (ug/dL)	12	59 - 158
Transferrin (mg/dL)	116	200 - 360
Total iron-binding capacity (µg/dL)	143	250 - 400

**Figure 1:**
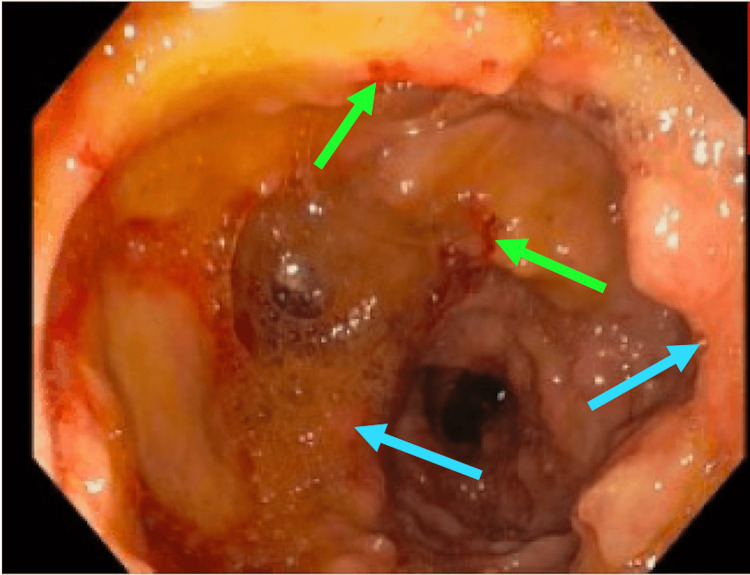
Visualization of the Jejunum With Push Enteroscopy Patchy erythematous mucosa (blue arrows) with evidence of active bleeding (green arrows).

A capsule endoscopy was performed, which identified patchy erythematous mucosa in the jejunum, which led to suspicion for Crohn’s Disease (CD) and further workup with push enteroscopy. Subsequent push enteroscopy identified patchy moderate mucosal changes characterized by nodularity and congestion in the second portion of the duodenum, and patchy erythematous mucosa with active bleeding in the proximal and mid jejunum (Figure 1). Several jejunal biopsies were obtained from areas with mucosal changes characterized by congestion and hemorrhage.

**Figure 2:**
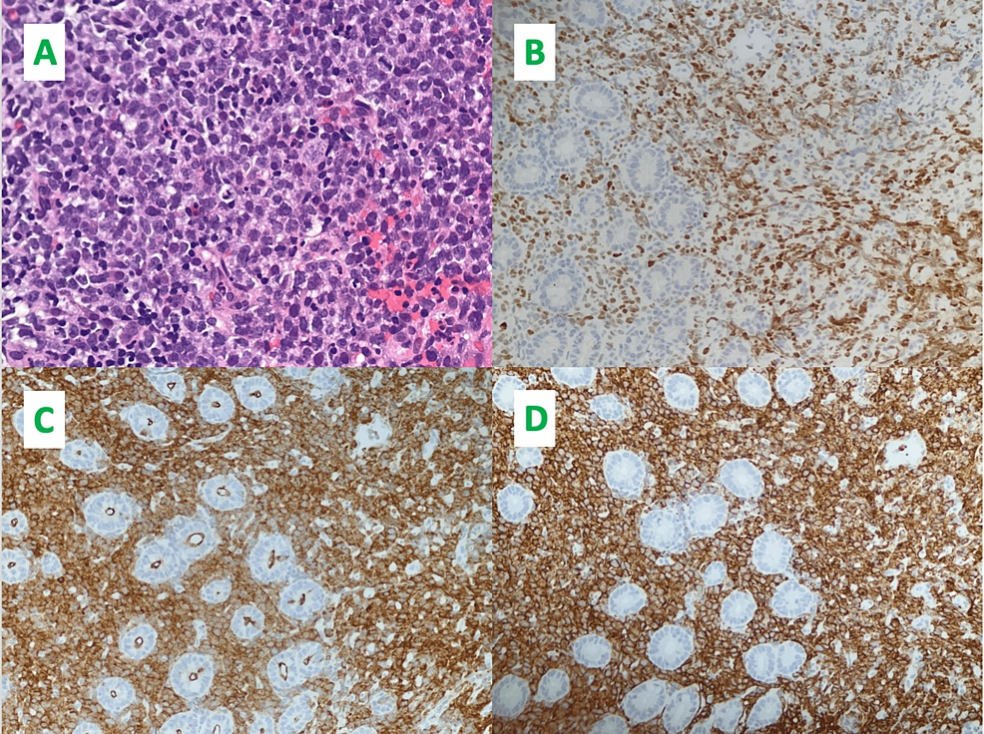
Pathology of Biopsy Revealing Diffuse Large B-cell Lymphoma Panel A: Hematoxylin and eosin (H&E) stain. Panel B: Immunohistochemistry (brown) detection of Bcl-6. Panel C: Immunohistochemistry (brown) detection of CD10. Panel D: Immunohistochemistry (brown) detection of CD20. All images are at 40x magnification.

Repeat colonoscopy demonstrated a diffuse area of moderately erythematous mucosa in the recto-sigmoid colon, and moderately congested mucosa in the proximal ascending colon. The terminal ileum appeared normal and was patent with no signs of inflammation or ulcers. Fecal calprotectin was found to be elevated to 2,893 ug/g and C-reactive protein was 219 mg/L. Biopsies eventually revealed diffuse large B-cell lymphoma (DLBCL) of the jejunum and focal active colitis with crypt abscess of the ascending colon. Immunohistochemical stains of the DLBCL demonstrated lymphoid cells positive for CD45, CD20, CD10, Bcl-6, and Mum-1, focally positive for C-myc, and negative for Bcl-2. The hematoxylin and eosin stain (H&E) and immunohistochemical detection of Bcl-6, CD10, and CD20 are displayed in Figure 2.

The patient was initiated on rituximab, cyclophosphamide, doxorubicin hydrochloride, vincristine sulfate, and prednisone combination therapy. Treatment for CD was deferred as the lymphoma was thought to be the primary etiology for the patient’s symptomatology. The patient was discharged to a subacute rehabilitation center for further care. Six months later, the patient had completed six cycles of chemotherapy. The patient denied any further weight loss, hematochezia, melena, subjective fevers, or chills. Laboratory results after six months demonstrated the patient’s hemoglobin levels rose, and remained stable between 10.3 g/dL and 10.9 g/dL.

## Discussion

Lymphomas represent only 5% to 10% of all gastrointestinal malignancies [5]. While gastrointestinal lymphomas are rare, between 70% to 95% of them are DLBCL [5]. Following the gastric tissue, the small bowel is the second most common location of a primary gastrointestinal lymphoma, seen in 20% to 35% of cases [6]. The five-year survival rate of intestinal lymphoma ranges from 33% to 55%, emphasizing the importance of early detection [7].

The presentation of small bowel lymphoma has historically caused delays in its diagnosis due to its presentation sometimes sharing many features, including symptomatology, roentgenograms, gross and microscopic findings with IBD, leading to misdiagnoses [8-14]. As seen in Table 2, there have been six previously reported cases of small bowel lymphomas initially misdiagnosed as CD. The delay in diagnosis of the lymphoma ranged from one month to 60 months and an average delay of 16.6 months (Table 2). All of these cases share at least some overlapping symptoms with other cases including abdominal discomfort, weight loss, generalized weakness, and blood loss (Table 2).

**Table 2:** All Published Cases in English of Small Bowel Lymphomas Initially Diagnosed as Crohn’s Disease

Article	Age	Sex	Symptoms	Months before lymphoma diagnosis	Lymphoma type	Lymphoma location	Involvement of ileum?
Chugh et al. [8]	68	Male	Abdominal pain, bloating, diarrhea, weight loss	18	B-cell lymphoma	Terminal ileum	yes
Chugh et al. [8]	76	Male	Abdominal pain, distention, small bowel obstruction	24	Low-grade follicular lymphoma	Mid-jejunum	yes
Kang et al. [12]	63	Female	Diarrhea, generalized weakness, weight loss	4	Angioimmunoblastic T-cell lymphoma	Terminal ileum	yes
Erkan et al. [15]	49	Male	Abdominal pain, nausea, vomiting, weight loss	1	Burkitt’s lymphoma	Terminal ileum	yes
Hurlstone [16]	50	Male	Anemia, diarrhea, weight loss	3	Mantle cell lymphoma	Terminal ileum	yes
Stundiene et al. [9]	50	Male	Abdominal pain, malaise, melena, weight loss	60	MALT lymphoma	Jejunum	yes
Our case	49	Male	Fatigue, melena, palpitations, weight loss	6	Diffuse Large B-cell lymphoma	Jejunum	no

While intestinal inflammation in CD can occur anywhere along the gastrointestinal tract, CD in most circumstances involves the ileum [17]. In patients with CD, 70% of cases are seen to involve the ileum [17]. This case stands out though given the confirmed absence of ileal involvement. For CD, there is evidence demonstrating increased re-operation rates and increased frequency of strictures in patients with jejunal CD compared to ileocecal CD [18,19]. While the applicability of this jejunal CD data as it relates to jejunal lymphoma mimicking jejunal CD remains unclear, given the apparent similarities between these two disease states, the aforementioned outcomes data for increased risks for strictures and recurrent operations may have a role in determining prognosis.

It is highly unlikely CD was present in this patient given the resolution of the patient’s symptoms, the improvement of the anemia, and the stability of the patient’s weight in the absence of treatment for CD. In addition, while the biopsies of the ascending colon demonstrated crypt abscess and colitis, histological finding that can appear in CD, cryptitis, crypt distortions and colitis have been described in prior cases of lymphoma mimicking IBD [9,12]. Also, prior literature has established an association between CD and adenocarcinoma, but whether CD is a risk factor for lymphoma, in the absence of immunomodulating therapy, remains controversial [3,20].

## Conclusions

Based on the historical delay of diagnosis, possible jejunal CD complications, and baseline high mortality, jejunal lymphoma mimicking CD has several risk factors for a poor outcome. While this is the first case documented in English of small bowel lymphoma mimicking CD without ileal involvement, and the third case mimicking CD with a primary jejunal lymphoma, it is possible there may be additional undiagnosed cases. Patients may be misdiagnosed with CD for years given the overlap of symptomatology between small bowel lymphoma and CD. Keeping intestinal malignancy on the differential in IBD patients with difficult-to-control disease states may ultimately help facilitate an earlier diagnosis of atypically presenting lymphoma. Practitioners in the future can consider ordering serum tumor markers during the IBD workup in order to screen for atypically presenting lymphoma, which may allow for an earlier identification if it is present.
